# Integrating Serum Lipid Biomarkers Into Machine Learning for the Differential Diagnosis of Breast Nodules

**DOI:** 10.1155/tbj/2642494

**Published:** 2026-08-21

**Authors:** Longmei Chen, Yuzhen Du, Wanchao Liu

**Affiliations:** ^1^ Department of Laboratory Medicine, Shanghai Baoshan Hospital of Integrated Traditional Chinese and Western Medicine, Baoshan Hospital, Shanghai University of Traditional Chinese Medicine, Shanghai 201999, China, shutcm.edu.cn; ^2^ Department of Laboratory Medicine, Shanghai Sixth People’s Hospital Affiliated to Shanghai Jiao Tong University School of Medicine, Shanghai 200233, China, sjtu.edu.cn

**Keywords:** breast cancer, machine learning, model interpretability, predictive model, serum lipid biomarkers

## Abstract

**Objective:**

To develop and validate an interpretable machine learning (ML) model for predicting malignant risk in patients with breast nodules using serum lipid biomarkers.

**Methods:**

This retrospective study included 899 patients with breast nodules (236 malignant) admitted between March 2022 and December 2024. Patients were randomly assigned to a training cohort (*n* = 630) and an internal validation cohort (*n* = 269) at a 7:3 ratio. Baseline clinical and laboratory data were collected upon admission. Following feature selection via LASSO regression, the predictive performance of 8 ML algorithms was evaluated and compared using receiver operating characteristic (ROC) curves. The optimal model’s performance was further corroborated using an independent temporal validation cohort of 190 patients (admitted Jan–Aug 2025). Model interpretability was addressed using SHapley Additive exPlanations (SHAP).

**Results:**

Nine key predictors were identified from 20 candidates by Lasso regression and clinical expertise. The random forest (RF) model outperformed other algorithms, achieving areas under the curve (AUC) values of 0.789, 0.782, and 0.825 for the training, internal validation, and temporal validation cohorts, respectively. Hosmer–Lemeshow tests (*p* > 0.05) indicated high calibration between the predicted and observed risks. SHAP importance analysis revealed Fer, age, and CEA to be the top three predictive factors.

**Conclusion:**

The RF model based on serum lipid biomarkers serves as a robust, noninvasive tool for assessing breast cancer risk, showing significant potential for clinical decision support in screening programs.

## 1. Introduction

Breast cancer (BC) remains the most prevalent malignancy and a leading cause of cancer‐related mortality among women under the age of 50 [1–3]. In 2024, China reported approximately 369,769 new cases and 77,959 deaths associated with BC [3]. Given the significant disease burden, accurate diagnosis and intervention are critical for reducing mortality and improving long‐term clinical outcomes [4, 5]. Consequently, there is an urgent clinical imperative to develop robust and accessible screening strategies for detection [6].

Despite the clinical dominance of mammography and ultrasonography as primary screening tools, their efficacy remains hampered by intrinsic limitations. Mammography, for instance, yields a suboptimal sensitivity (65%) for early‐stage malignancies and poses concerns regarding ionizing radiation exposure [7–9]. While ultrasonography compensates for these gaps in dense breast tissues—a prevalent phenotype in Asian women—it is often plagued by poor specificity, culminating in excessive false positives and unwarranted invasive procedures [10, 11]. To overcome these limitations, machine learning (ML) has been harnessed to refine risk stratification. Nevertheless, the geographic bias inherent in models calibrated on Western cohorts frequently leads to risk overestimation in Asian populations [12–14]. Furthermore, while earlier versions relied heavily on demographic and conventional imaging features, more recent models incorporating genetic variants or immune‐related biomarkers are often constrained by high costs and methodological complexity, limiting their widespread clinical utility [15–20].

Emerging evidence underscores the pivotal role of lipid metabolism in the pathogenesis of BC. The dysregulation of enzymes and regulatory factors involved in lipid biosynthesis and catabolism significantly influence the proliferation and metastasis of breast cancer cells (BCCs) [21]. In the context of BC, lipid metabolism functions not only as an essential energy reservoir for tumor cells but also as a trigger for specific oncogenic signaling pathways and aberrant cellular activities [22]. Experimental murine models have confirmed that hyperlipidemia can drive tumor growth [23]. Clinically, circulating low‐density lipoprotein cholesterol (LDL‐C) levels have been identified as predictive markers for breast tumor progression [24]—a finding further corroborated by Mendelian randomization studies suggesting a causal link between genetically elevated LDL‐C and increased BC incidence. Additionally, retrospective data indicate that lower high‐density lipoprotein cholesterol (HDL‐C) levels are associated with poorer overall survival, while increased triglycerides (TG) and cholesterol synthesis facilitate tumor migration and metastasis [21]. Despite the established link between lipid dysmetabolism and BC, the integration of comprehensive lipid profiles into predictive modeling remains underdeveloped. While novel lipid‐derived indices—such as the PLT–HDL‐C ratio (PHR) [25], TC–HDL‐C ratio [26], lymphocyte–HDL‐C ratio (LHR), and CRP–HDL‐C ratio [27]—have demonstrated diagnostic value in metabolic disorders, indices such as the neutrophil‐to‐HDL ratio (NHR) [28] and monocyte‐to‐HDL ratio (MHR) [29] have only recently been recognized as independent diagnostic factors for BC.

In this study, we aimed to address these gaps from a metabolomic perspective by developing and validating an ML‐based risk assessment model for malignant breast nodules. By integrating serum lipid profiles, derived metabolic indices, and conventional tumor markers, we sought to provide a cost‐effective, noninvasive, and highly accurate auxiliary tool tailored for the clinical management of BC in the Chinese population.

## 2. Materials and Methods

### 2.1. Study Design

**FIGURE 1:**
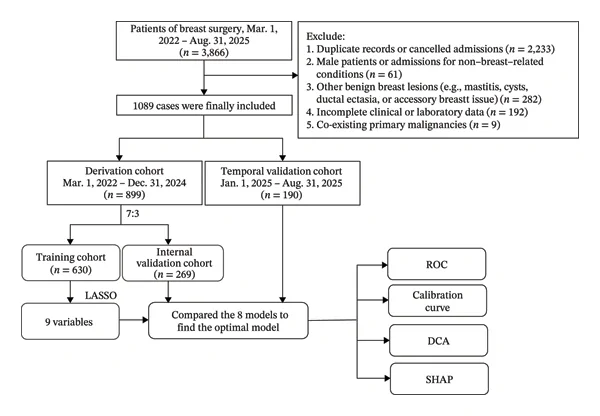
Flowchart of patient enrollment and study design. Abbreviations: LASSO, least absolute shrinkage and selection operator; ROC, receiver operating characteristic; DCA, decision curve analysis; SHAP, SHapley Additive exPlanations.

The overall objective was to develop an interpretable ML model for differentiating malignant from benign breast nodules using routinely available clinical and laboratory variables. The workflow (Figure 1) included cohort construction, feature selection, model development, independent validation, and model interpretation. First, 70% of the derivation cohort was used as the training cohort to develop predictive models, with the remaining 30% serving as the internal validation cohort. An independent dataset from our hospital was used as a temporal validation cohort. The model’s performance was evaluated based on the pathological results. The SHapley Additive exPlanations (SHAP) algorithm was employed to clarify the significance of features in the predictive model and identify the interactions among risk prediction variables.

### 2.2. Study Participants

The derivation and temporal validation cohorts followed identical inclusion and exclusion criteria. Inclusion criteria were (i) female patients with pathologically confirmed benign fibroadenoma or malignant BC; (ii) newly diagnosed cases with no prior history of breast‐related surgery, radiotherapy, or chemotherapy; and (iii) availability of complete clinical, laboratory, and pathological records. Exclusion criteria were (i) male patients; (ii) other benign breast lesions (e.g., mastitis, cysts, ductal ectasia, or accessory breast tissue); (iii) insufficient pathological or laboratory data; and (iv) coexisting primary malignancies in other organs (Figure 1).

Retrospective medical records of patients treated at the Department of Breast Surgery in our hospital from March 2022 to August 2025 were screened. Among the initially retrieved 3866 patients, we excluded individuals with repeated admissions (*n* = 2233), non–breast diseases (*n* = 61), other benign breast lesions (*n* = 282), missing laboratory or clinical data (*n* = 192), and concurrent malignant tumors other than BC (*n* = 9). Patient identity information was cross‐referenced across datasets to eliminate duplicate participants between different analytical cohorts.

Finally, a total of 1089 eligible patients were enrolled. Among them, 899 patients diagnosed from March 2022 to December 2024 formed the derivation cohort, consisting of 236 malignant and 663 benign cases. The remaining 190 patients diagnosed between January 2025 and August 2025 constituted the temporal validation cohort, including 51 malignant and 139 benign cases.

### 2.3. Data Collection and Clinical Definitions

Clinical and laboratory data were retrospectively extracted from the electronic medical record (EMR) and laboratory information system (LIS). Fasting venous blood samples were collected from all patients at admission before any clinical intervention.

Complete blood counts (CBCs) were performed after a 30‐minute room‐temperature incubation. Specimens were centrifuged at 3500 × g for 10 min, and serum samples were processed for subsequent detection within 4 h after collection. Whole blood parameters were determined using a Sysmex XN‐9000 automatic hematology analyzer (Sysmex Corp., Kobe, Japan), namely, neutrophils (NE) count, lymphocytes (LY) count, monocytes (MO) count, and platelet (PLT) count. High‐sensitivity C‐reactive protein (hs‐CRP) levels were assessed via a Genrui PA200 specific protein analyzer (Genrui Biotech Co., Ltd., Shenzhen, China). Serum lipid profiles, including total cholesterol (TC), TG, HDL‐C, and LDL‐C, were detected using a Beckman Coulter AU5800 automatic biochemical analyzer. Serum tumor markers, namely ferritin (Fer), alpha‐fetoprotein (AFP), carcinoembryonic antigen (CEA), carbohydrate antigen 199 (CA199), carbohydrate antigen 125 (CA125), carbohydrate antigen 153 (CA153), carbohydrate antigen 50 (CA50), carbohydrate antigen 724 (CA724), and squamous cell carcinoma (SCC) antigen, were quantified via the chemiluminescence immunoassay method on a Beckman Coulter DXI800 immunoassay system (Beckman Coulter, Inc., Brea, CA, USA).

Based on the routine laboratory parameters, several lipid‐inflammatory composite indices were calculated for predictive model construction: platelet‐to‐HDL‐C ratio (PHR) = PLT/HDL‐C, TC‐to‐HDL‐C ratio (TC–HDL‐C) = TC/HDL‐C, lymphocyte‐to‐HDL‐C ratio (LHR) = LY/HDL‐C, monocyte‐to‐HDL‐C ratio (MHR) = MO/HDL‐C, neutrophil‐to‐HDL‐C ratio (NHR) = NE/HDL‐C, and hs‐CRP‐to‐HDL‐C ratio (CRP–HDL‐C) = hs‐CRP/HDL‐C [28].

Hyperlipidemia was defined as the presence of at least one lipid abnormality based on established guidelines: TC ≥ 5.2 mmol/L, TG ≥ 1.7 mmol/L, LDL‐C ≥ 3.4 mmol/L, or HDL‐C < 1.0 mmol/L [30]. Given the unavailability of detailed menstrual records in this retrospective cohort, postmenopausal status was approximated using an age cutoff of 50 years or older.

### 2.4. Variable Selection

Within the derivation cohort, patients were randomly assigned to a training cohort (*n* = 630, 167 malignant) and an internal validation cohort (*n* = 269, 69 malignant) at a 7:3 ratio. Prior to feature selection and model construction, missing data in the training, internal, and temporal validation cohorts were separately imputed using the multiple imputation by chained equations (MICEs) approach with a random forest (RF) conditional model.

Least absolute shrinkage and selection operator (LASSO) regression was selected because it simultaneously performs feature selection and coefficient shrinkage, thereby reducing model complexity and minimizing overfitting. All continuous variables were standardized via Z‐score normalization. LASSO regression with 10‐fold cross‐validation was performed solely within the training cohort to screen optimal predictive features; variables with nonzero coefficients at the lambda.1se criterion were retained as independent predictors for the final predictive model. Subsequently, variance inflation factor (VIF) analysis was conducted to assess multicollinearity.

### 2.5. Model Development and Evaluation

Predictive models were constructed within the Tidymodels framework in R. Eight ML algorithms were then implemented and compared: Logistic regression (Logistic), Naive Bayes (NB), RF, support vector machine (SVM), extreme gradient boosting (XGBoost), decision tree (DT), K‐nearest neighbor (KNN), and multilayer perceptron (MLP).

Hyperparameter calibration for RF and XGBoost was carried out via Latin hypercube sampling within prespecified search ranges. For RF, the tuned hyperparameters encompassed mtry (3–10), tree count (100–800), and minimum node size (2–15), with 15 distinct parameter combinations assessed. XGBoost underwent an identical sampling scheme to optimize the number of trees (100–800), learning rate (0.01–0.30), maximum tree depth (3–10), and minimum node size (2–15).

All hyperparameter searches were executed under stratified 5‐fold cross‐validation, with the optimal parameter configuration chosen according to the highest mean area under the receiver operating characteristic curve (ROC–AUC). To enable unbiased head‐to‐head comparisons among algorithms, logistic, NB, SVM, DT, KNN, and MLP models were trained with default hyperparameters supplied by their corresponding R packages. After identifying optimal hyperparameters, each algorithm was refitted on the complete training dataset to produce fixed, locked models for subsequent validation across internal and temporal validation cohorts. The optimal binary classification cutoff was determined by maximizing the Youden index from the receiver operating characteristic (ROC) curves, and then this unified threshold was adopted across the validation cohorts to derive categorical labels for performance quantification.

SHAP was chosen because it provides both global and local explanations and enables interpretation of complex nonlinear ML models. SHAP values were calculated exclusively for the best‐performing RF model using the R fastshap package. A model‐agnostic SHAP framework was adopted to quantify each feature’s marginal contribution to the predicted risk of malignancy. A customized prediction wrapper was constructed to extract class‐specific malignancy probabilities from the ranger model outputs. A total of 100 Monte Carlo replicates (nsim = 100) were used to compute SHAP values, striking a balance between computational efficiency and robust estimation. Global interpretability metrics and local feature‐response relationships were both derived from SHAP outputs, where overall feature importance was quantified as the mean absolute SHAP value across all samples. The final optimized RF model selected via hyperparameter tuning was refitted with 500 trees for SHAP interpretation.

### 2.6. Statistical Analysis

Statistical analyses were performed using R (Version 4.5.1) and Python (Version 3.14.2). Data normality was assessed using the Shapiro–Wilk test. Continuous variables were expressed as mean ± standard deviation (SD) for normally distributed data and compared via independent‐sample Student′s *t*‐tests. Nonnormally distributed data were presented as medians (interquartile range [IQR]) and analyzed using the Mann–Whitney *U* test. Categorical variables were expressed as frequencies (percentages) and compared using the chi‐square test. A two‐tailed *p* < 0.05 was considered statistically significant.

## 3. Results

### 3.1. Baseline Clinical Characteristics in the Derivation Cohort

Normality testing indicated that all continuous variables followed a nonnormal distribution; thus, data were presented as medians with IQRs [M (Q1, Q3)]. Comparisons between the benign and malignant groups revealed that patients with malignancies were significantly older and exhibited higher levels of TC, TG, LDL‐C, TC–HDL‐C, LHR, CRP–HDL‐C, Fer, CEA, and CA153 (*p* < 0.05). Conversely, HDL‐C, CA199, CA125, and SCC levels were significantly lower in the malignant group (*p* < 0.05; Table 1). The proportion of postmenopausal patients was significantly higher in the malignant group (66.95% vs. 24.13%, *p* < 0.001). Furthermore, the prevalence of hyperlipidemia was significantly higher in patients with malignant nodules compared to those with benign lesions (53.39% vs. 38.91%, *p* < 0.001).

**TABLE 1:** Baseline characteristics of patients with benign and malignant breast nodules in the derivation cohort.

Variables	Total (*n* = 899)	Benign nodules (*n* = 663)	Malignant nodules (*n* = 236)	*p*
Age, M (Q_1_, Q_3_)	45.00 (37.00, 57.00)	42.00 (34.00, 50.00)	57.00 (48.00, 64.00)	< 0.001
Menopausal status, *n* (%)				< 0.001
No	581 (64.63)	503 (75.87)	78 (33.05)	
Yes	318 (35.37)	160 (24.13)	158 (66.95)	
TC, M (Q_1_, Q_3_)	4.98 (4.35, 5.58)	4.91 (4.33, 5.51)	5.14 (4.43, 5.87)	0.012
TG, M (Q_1_, Q_3_)	1.00 (0.75, 1.42)	0.93 (0.69, 1.33)	1.22 (0.90, 1.73)	< 0.001
HDL‐C, M (Q_1_, Q_3_)	1.44 (1.24, 1.65)	1.45 (1.25, 1.66)	1.38 (1.19, 1.57)	0.005
LDL‐C, M (Q_1_, Q_3_)	3.05 (2.60, 3.53)	2.98 (2.58, 3.45)	3.25 (2.72, 3.77)	< 0.001
PHR, M (Q_1_, Q_3_)	163.92 (128.99, 200.00)	163.13 (125.72, 199.70)	164.55 (131.69, 200.04)	0.484
TC–HDL‐C, M (Q_1_, Q_3_)	3.42 (2.98, 4.02)	3.34 (2.89, 3.88)	3.66 (3.17, 4.31)	< 0.001
LHR, M (Q_1_, Q_3_)	1.10 (0.88, 1.52)	1.09 (0.88, 1.45)	1.17 (0.92, 1.66)	0.016
MHR, M (Q_1_, Q_3_)	0.25 (0.19, 0.34)	0.25 (0.19, 0.33)	0.27 (0.20, 0.35)	0.080
NHR, M (Q_1_, Q_3_)	2.45 (1.84, 3.26)	2.42 (1.80, 3.20)	2.57 (1.94, 3.36)	0.050
CRP–HDL‐C, M (Q_1_, Q_3_)	0.41 (0.33, 0.83)	0.39 (0.32, 0.75)	0.47 (0.34, 1.31)	< 0.001
Fer, M (Q_1_, Q_3_)	41.20 (16.70, 91.70)	31.95 (14.20, 72.53)	84.40 (37.90, 152.90)	< 0.001
AFP, M (Q_1_, Q_3_)	2.75 (1.99, 3.93)	2.76 (2.00, 3.94)	2.68 (1.96, 3.90)	0.524
CEA, M (Q_1_, Q_3_)	1.05 (0.56, 1.83)	0.90 (0.50, 1.41)	1.62 (0.97, 2.73)	< 0.001
CA199, M (Q_1_, Q_3_)	9.29 (5.00, 17.13)	9.86 (5.25, 17.38)	7.96 (4.16, 15.44)	0.035
CA125, M (Q_1_, Q_3_)	11.03 (7.97, 15.56)	11.39 (8.29, 16.27)	10.38 (7.40, 13.13)	< 0.001
CA153, M (Q_1_, Q_3_)	10.33 (8.11, 16.48)	10.15 (8.01, 15.95)	11.18 (8.69, 17.64)	0.047
CA50, M (Q_1_, Q_3_)	5.06 (3.09, 7.46)	4.95 (3.02, 7.36)	5.27 (3.39, 7.90)	0.154
CA724, M (Q_1_, Q_3_)	1.79 (0.85, 3.46)	1.77 (0.83, 3.52)	1.85 (0.92, 3.19)	0.768
SCC, M (Q_1_, Q_3_)	1.04 (0.76, 1.34)	1.07 (0.79, 1.36)	0.92 (0.72, 1.24)	0.001
Hyperlipidemia, *n* (%)				< 0.001
No	515 (57.29)	405 (61.09)	110 (46.61)	
Yes	384 (42.71)	258 (38.91)	126 (53.39)	

### 3.2. Feature Selection and Variable Importance

**FIGURE 2:**
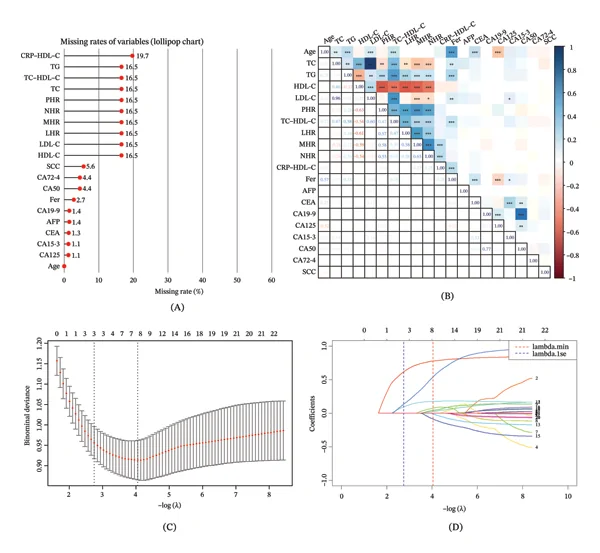
Variable correlation and feature selection using LASSO regression. (A) Lollipop plot of missingness rates for candidate variables. (B) Heatmap of Spearman correlation coefficients between variables. (C) Selection of the optimal tuning parameter (lambda) via 10‐fold cross‐validation. (D) LASSO coefficient profiles of the 20 candidate variables.

Missingness analysis of the 20 candidate variables in the training cohort (Figure 2A) identified CRP–HDL‐C as the feature with the highest missing rate (19.7%). After imputing by MICE, correlation analysis (Figure 2B) revealed substantial collinearity among lipid‐related parameters. To mitigate redundancy and enhance model interpretability, LASSO regression was employed for dimensionality reduction, yielding eight key variables: age, TC–HDL‐C, MHR, CRP–HDL‐C, Fer, AFP, CEA, and CA199 (Figure 2C,D). Although CA153 was not retained by the LASSO algorithm, it was incorporated because it is one of the most widely used serum biomarkers for BC surveillance and has well‐established clinical relevance. This strategy was intended to preserve clinically meaningful information while maintaining model interpretability. The VIF values of all nine predictors were less than 2, indicating negligible multicollinearity among the variables (Supporting Table S1). Univariate logistic regression analysis of nine predictive variables was listed in Table 2. The MHR exhibited the highest odds ratio (OR) of 4.69 (95% CI: 1.08–20.41). Each one‐unit increment in MHR was associated with an elevated risk of malignant breast lesions relative to benign breast nodules. Age, Fer, and CEA also demonstrated significant associations with malignant breast nodules. These findings were generally consistent with the variables identified by the LASSO algorithm, supporting the robustness of the selected predictors.

**TABLE 2:** Univariate logistic regression analysis of predictors associated with malignant lesions.

Variables	*β*	SE	*Z*	*p*	OR (95% CI)
Age	0.09	0.01	10.03	< 0.001	1.09 (1.07 ∼ 1.11)
MHR	1.55	0.75	2.06	0.039	4.69 (1.08 ∼ 20.41)
TC–HDL‐C	0.56	0.13	4.39	< 0.001	1.75 (1.36 ∼ 2.24)
CRP–HDL‐C	0.11	0.05	2.40	0.016	1.11 (1.02 ∼ 1.22)
AFP	−0.03	0.05	−0.63	0.527	0.97 (0.87 ∼ 1.07)
CEA	0.70	0.09	7.44	< 0.001	2.01 (1.67 ∼ 2.41)
CA19‐9	−0.01	0.01	−0.96	0.335	0.99 (0.98 ∼ 1.01)
Fer	0.01	0.00	7.92	< 0.001	1.01 (1.01 ∼ 1.01)
CA15‐3	0.02	0.01	1.95	0.051	1.02 (1.00 ∼ 1.04)

*Note: β*, regression coefficient; *Z*, Z statistic; *p*, *p* value.

Abbreviations: CI, confidence interval; OR, odds ratio; SE, standard error.

### 3.3. Model Development

**FIGURE 3:**
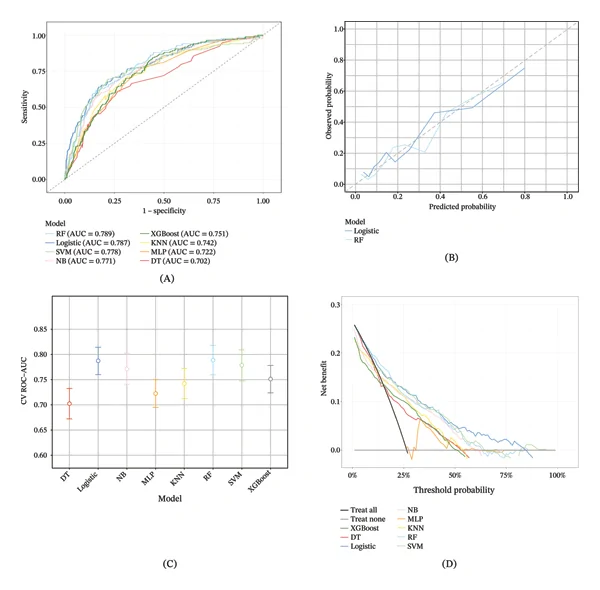
Performance evaluation of eight machine learning models in the training cohort. (A) ROC curves for eight models. (B) Calibration curves showing the agreement between predicted and observed probabilities. (C) Point plot of AUC values across 5‐fold cross‐validation. (D) Decision curve analysis (DCA) evaluating the clinical net benefit of the models.

The selected nine variables were incorporated into 8 ML algorithms. In the training cohort, the RF model achieved the highest discriminative performance (AUC = 0.789, 95% CI: 0.731–0.846), followed by logistic (AUC = 0.787, 95% CI: 0.734–0.840) (Figure 3A, Table 3). Fivefold cross‐validation (Figure 3B) demonstrated consistent stability, with mean AUCs ranging from 0.70 to 0.78, among which RF and Logistic remained the top performers. The Hosmer–Lemeshow (HL) test indicated no significant lack of fit (*χ* ^2^ = 11.28, *p* = 0.19; Figure 3C), signifying strong calibration. Decision curve analysis (DCA) demonstrated that across the threshold probability range of 0.1–0.8, all models provided superior net benefits compared to “intervention‐for‐all” or “intervention‐for‐none” strategies (Figure 3D). Compared with other algorithms, the RF model demonstrated a better balance between discrimination, calibration, and clinical utility, and was therefore selected for subsequent validation and interpretation.

**TABLE 3:** Evaluation parameters of 8 models on the training cohort.

Model	AUC	95% CI	Sensitivity (%)	Specificity (%)	PPV	NPV	Accuracy (%)	Youden index	Brier
RF	0.789	0.731–0.846	61.1	84.2	0.58	0.86	78.1	0.45	0.15
Logistic	0.787	0.734–0.840	63.5	82.9	0.57	0.86	77.8	0.46	0.15
SVM	0.778	0.718–0.838	68.9	78.2	0.53	0.87	75.7	0.47	0.15
NB	0.771	0.710–0.832	67.1	75.0	0.49	0.86	72.9	0.42	0.18
XGBoost	0.751	0.698–0.804	83.8	55.7	0.41	0.91	63.2	0.40	0.20
KNN	0.742	0.684–0.801	64.1	74.1	0.47	0.85	71.4	0.38	0.19
MLP	0.722	0.669–0.776	70.7	66.3	0.43	0.86	67.5	0.37	0.19
DT	0.702	0.643–0.762	57.5	75.0	0.45	0.83	70.3	0.32	0.19

*Note:* AUC, area under the receiver operating characteristic curve; NPV, negative predictive value; PPV, positive predictive value.

Abbreviations: CI, confidence interval; DT, decision tree; KNN, K‐nearest neighbor; Logistic, logistic regression; MLP, multilayer perceptron; NB, Naive Bayes; NPV, negative predictive value; PPV, positive predictive value; RF, random forest; SVM, support vector machine; XGBoost, extreme gradient boosting.

### 3.4. Internal and Temporal Validation

**FIGURE 4:**
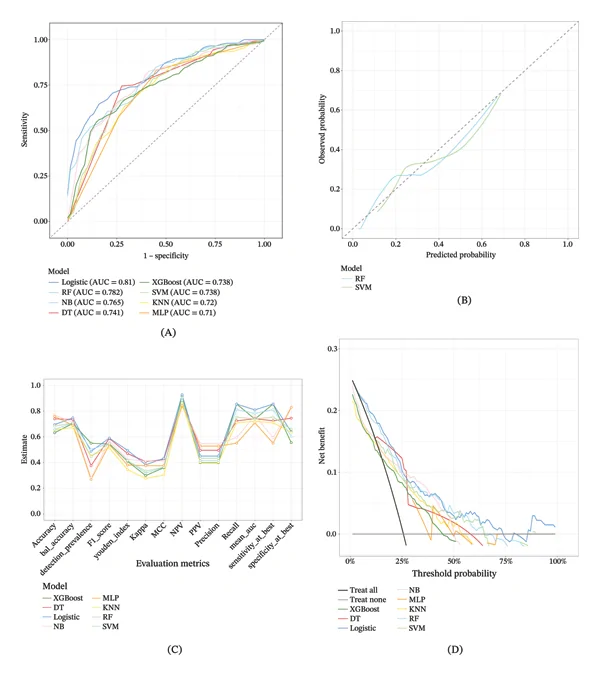
Performance evaluation and internal validation of eight machine learning models. (A) ROC curves for eight models in the internal validation cohort. (B) Calibration curves showing the agreement between predicted and observed probabilities. (C) Line chart of multiple performance metrics (e.g., accuracy, sensitivity, and specificity). (D) Decision curve analysis (DCA) comparing the clinical net benefit of the eight models.

In the internal validation cohort, the RF model maintained robust discriminative power with an AUC of 0.782 (95% CI: 0.767–0.798) (Figure 4A). The HL test (*χ* ^2^ = 1.27, *p* = 0.26) indicated excellent calibration between predicted and observed risks (Figure 4B). Multiple performance metrics, including AUC, optimal cutoff value, sensitivity, specificity, accuracy, F1‐score, Brier score, positive/negative predictive values (PPV/NPV) and the Youden Index, are visualized in Figure 4C and Table 4. DCA further confirmed a significant net benefit within the 0–0.7 threshold range (Figure 4D). The comparable performance observed in the internal validation cohort suggested that the RF model maintained good generalizability without evident overfitting.

**TABLE 4:** Evaluation parameters of 8 models on the internal validation cohort.

Model	AUC	95% CI	Sensitivity (%)	Specificity (%)	PPV	NPV	Accuracy (%)	Youden index	Brier
XGBoost	0.738	0.723–0.753	85.5	55.5	0.40	0.92	63.2	0.41	0.21
DT	0.741	0.725–0.756	72.5	74.5	0.50	0.89	74.0	0.47	0.17
Logistic	0.810	0.795–0.825	85.5	64.0	0.45	0.93	69.5	0.50	0.15
NB	0.765	0.750–0.780	59.4	83.0	0.55	0.86	77.0	0.42	0.18
MLP	0.710	0.695–0.725	55.1	83.0	0.53	0.84	75.8	0.38	0.19
KNN	0.720	0.705–0.736	71.0	63.5	0.40	0.86	65.4	0.35	0.19
RF	0.782	0.767–0.798	81.2	60.5	0.41	0.90	65.8	0.42	0.16
SVM	0.738	0.723–0.753	75.4	66.0	0.43	0.89	68.4	0.41	0.17

**FIGURE 5:**
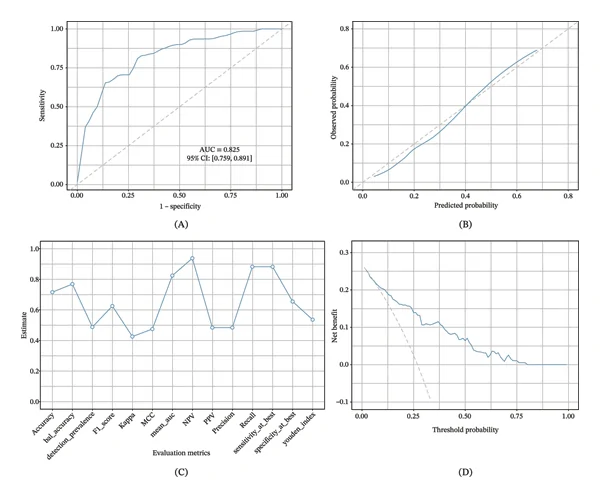
Performance of the random forest (RF) model in the temporal validation cohort. (A) ROC curve showing discriminative power. (B) Calibration curve showing the consistency between predicted and actual outcomes. (C) Multiple performance metrics for the RF model. (D) Decision curve analysis (DCA) demonstrating clinical utility.

Furthermore, the RF model also demonstrated enhanced performance with an AUC of 0.825 (95% CI: 0.759–0.891; Figure 5A) in the temporal validation cohort. Calibration remained strong (HL test: *χ* ^2^ = 12.72, *p* = 0.12; Figure 5B), and various performance parameters underscored the model’s reliability (Figure 5C), and DCA corroborated its clinical utility within the 0.0–0.7 threshold interval (Figure 5D). The preserved discrimination and calibration in the temporally independent cohort further support the robustness and potential clinical applicability of the proposed model.

### 3.5. Model Interpretation

**FIGURE 6:**
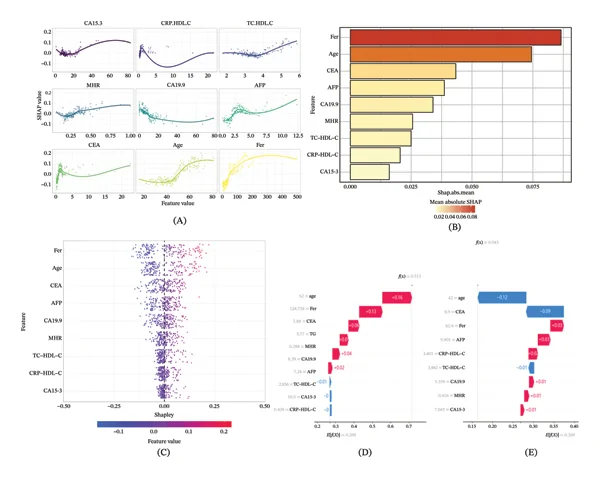
Model interpretability and SHAP analysis of the Random Forest (RF) model. (A) SHAP dependence plots illustrating the nonlinear relationships between predictors and outcomes. (B) SHAP feature importance ranking. (C) SHAP summary (beeswarm) plot showing the distribution of variable contributions across all samples. (D) SHAP waterfall plot for a representative positive (malignant) case. (E) SHAP waterfall plot for a representative negative (benign) case.

SHAP dependency plots revealed complex, nonlinear relationships between the nine variables and the risk of malignancy (Figure 6A). CA15‐3, TC–HDL‐C, MHR, CEA, age, and Fer showed overall positive correlations with malignant risk: Their SHAP values gradually rose as feature levels increased. In addition, AFP exhibited a biphasic trend, with elevated malignant risk only observed at high AFP concentrations. CA19‐9 exerted a protective effect, where higher levels corresponded to lower SHAP values and reduced odds of malignancy. On the other hand, the CRP–HDL‐C followed a U‐shaped curve, with the lowest malignant predictive contribution observed at intermediate values. All predictors demonstrated evident nonlinear effects on the model output, consistent with the direction of associations derived from multivariate logistic regression.

SHAP feature importance ranking identified Fer, age, and CEA as the top three predictors (Figure 6B). The SHAP summary plot (Figure 6C) illustrates the distribution of variable contributions across all samples, where higher values of Fer and age were positively associated with increased malignancy risk.

Individual case interpretations using SHAP waterfall plots further quantified these contributions. For a representative positive sample (Figure 6D), Fer (+0.13) and age (+0.16) exerted the strongest positive influence on the probability of malignancy. Conversely, for a negative sample (Figure 6E), age (−0.12) and CEA (−0.09) were the primary factors reducing the predicted risk. These findings validate that Fer and age are critical predictors for identifying malignant breast nodules.

Overall, SHAP analysis demonstrated that the RF model captured complex nonlinear interactions among serum biomarkers rather than relying on single‐variable effects. The consistency between SHAP rankings and conventional statistical analyses further enhanced the interpretability and credibility of the proposed model.

## 4. Discussion

In this study, we developed and validated a novel diagnostic model centered on lipid metabolism parameters for the identification of malignant breast nodules. Utilizing the RF algorithm, the model demonstrated robust discriminative performance (AUC = 0.789) by integrating age with eight routine hematological indices. Compared with conventional models based primarily on demographic or single biomarker information, our model integrates lipid metabolism‐related parameters with routine clinical indicators, providing a more comprehensive assessment of malignancy risk. Furthermore, the model’s stable performance in the temporal validation cohort (AUC = 0.825) and superior net benefit in DCA underscore its substantial potential for clinical translation.

Several methodological strategies were adopted to improve the robustness of the model, including pathology‐confirmed outcomes, multiple imputation for missing data, and biologically informed feature selection. To determine the optimal architecture, we systematically evaluated 8 ML algorithms. Following 5‐fold cross‐validation, the RF model emerged as the most robust architecture, achieving a superior balance between sensitivity and specificity. Unlike conventional regression models that assume linear relationships, the RF algorithm effectively captured complex nonlinear interactions among lipid metabolism, inflammatory status, and tumor‐associated biomarkers, which likely contributed to its superior diagnostic performance. Moreover, the enhanced AUC of 0.825 in the independent temporal cohort confirms the model’s generalizability, establishing it as a reliable high‐performance auxiliary tool for clinical BC screening. The slightly higher performance observed in the temporal validation cohort may reflect differences in cohort composition rather than model overfitting, further supporting the robustness of the proposed framework.

SHAP analysis identified Fer, CEA, AFP, CA199, and CA153 as the most influential tumor markers in the RF model. Elevated Fer in BC is closely linked to disease progression, as tumor‐associated macrophages can release ferritin, triggering inflammatory responses that facilitate tumorigenesis [31]. Consistent with these biological mechanisms, the prominent contribution of Fer in our model suggests that systemic iron metabolism plays a critical role in distinguishing malignant from benign breast nodules. The inclusion of CEA, CA199, and AFP supports the synergy of multimarker panels, which has been shown to surpass the diagnostic efficacy of single‐marker assays [32]. The high SHAP ranking of CEA further indicates that conventional tumor markers remain informative when combined with lipid metabolism‐related indicators rather than being used in isolation. Notably, CA153 serves not only as a high‐specificity diagnostic marker but also as a prognostic indicator, where higher levels correlate with poorer overall survival [33]. Furthermore, our findings reinforce that BC risk increases with age, particularly in postmenopausal women [34]. Among lipid‐related markers, MHR, TC–HDL‐C, and CRP–HDL‐C exerted the most significant impact. MHR is notably elevated in BC with lymphatic metastasis, offering predictive value for nodal involvement [28]. The TC–HDL‐C has recently been recognized as a superior indicator of metabolic derangement compared to individual lipid parameters [35]. Given that chronic low‐grade inflammation [28] and lipid dysregulation [23] are hallmarks of BC pathophysiology, our study demonstrates that CRP–HDL‐C serves as a potent composite marker reflecting both systemic inflammation and metabolic status. Collectively, these findings demonstrate that the RF model captures complementary biological information from tumor biomarkers, lipid metabolism, and inflammatory status, thereby enhancing both predictive performance and biological interpretability.

This study contributes two primary innovations to the field of BC diagnostics. First, we developed a predictive model by strategically integrating lipid metabolism indices—a domain that remains remarkably underexplored despite the known metabolic reprogramming in cancer. Second, by utilizing exclusively routine hematological parameters, we established a streamlined yet high‐performing framework that is highly accessible for resource‐limited clinical settings. Third, the incorporation of SHAP analysis enhanced the interpretability of the model by providing both global and individual‐level explanations, enabling clinicians to better understand how specific biomarkers contribute to each prediction.

Several limitations of this study warrant consideration. First, although temporal validation was performed, our cohort was recruited from a single institution; thus, external validation in geographically diverse populations is required. Second, the retrospective design precludes a definitive assessment of whether these hematological markers can facilitate earlier detection compared with conventional imaging modalities. Finally, while the current model provides a pragmatic foundation, future iterations should explore multimodal fusion by incorporating radiomic features or molecular signatures.

In conclusion, we successfully developed and validated a lipid metabolism‐oriented RF model. By leveraging routinely available serum lipid parameters, this model provides a robust and accessible tool for the differential diagnosis of malignant breast nodules. This model may provide an inexpensive and readily available adjunct to conventional imaging, thereby supporting individualized clinical decision‐making.

## Author Contributions

**Wanchao Liu**: conception and design of the study (lead); provision of the overall conceptual framework (lead); **Longmei Chen**: formal analysis (equal); data visualization (equal); writing–original draft (equal); **Yuzhen Du**: result validation (equal); writing–review and editing (equal).

## Funding

This work was supported by the Outstanding Young Medical Talents Training Program of Shanghai Baoshan Hospital of Integrated Traditional Chinese and Western Medicine (Baoshan Hospital, Shanghai University of Traditional Chinese Medicine) (No. 2023BY003).

## Ethics Statement

Ethical approval was granted by the Ethics Review Committee of Shanghai Baoshan Hospital of Integrated Traditional Chinese and Western Medicine (Baoshan Hospital, Shanghai University of Traditional Chinese Medicine) (Approval Number: 2023K42).

## Conflicts of Interest

The authors declare no conflicts of interest.

## Supporting Information

Additional supporting information can be found online in the Supporting Information section.

## Supporting information


**Supporting Information** Supporting Table S1. Variance inflation factor (VIF) values of the nine predictive variables in the training cohort. All VIF values were below 2, suggesting no obvious multicollinearity among these predictors.

## Data Availability

All raw data and code are available upon request.
