# Supplementary material for: Integrating Serum Lipid Biomarkers Into Machine Learning for the Differential Diagnosis of Breast Nodules
Source: Breast J. 2026 Aug 21;2026:2642494. doi: 10.1155/tbj/2642494 (PMC13495554; doi:10.1155/tbj/2642494)
Supplement: Supplementary file 1 [file TBJ-2026-2642494-s001.docx]

Supplementary Table S1. The VIF value of 9 variables in the training corhort

| Variables | age | TC-HDL-C | MHR | CRP-HDL-C | Fer | AFP | CEA | CA19-9 | CA15-3 |
| --- | --- | --- | --- | --- | --- | --- | --- | --- | --- |
| VIF value | 1.54 | 1.21 | 1.20 | 1.15 | 1.63 | 1.01 | 1.20 | 1.02 | 1.14 |
